# Modeling of mitochondrial bioenergetics and autophagy impairment in MELAS-mutant iPSC-derived retinal pigment epithelial cells

**DOI:** 10.1186/s13287-022-02937-6

**Published:** 2022-06-17

**Authors:** Sujoy Bhattacharya, Jinggang Yin, Weihong Huo, Edward Chaum

**Affiliations:** Department of Ophthalmology and Visual Sciences, Vanderbilt University Medical Center, 2311 Pierce Avenue, Nashville, TN 37232 USA

**Keywords:** Age-related macular degeneration, MELAS, iPSC-derived retinal pigment epithelium, Autophagy flux, Mitophagy, Mitochondrial heteroplasmy, Prom1/CD133, AMPKα, PGC-1α, Regenerative medicine

## Abstract

**Background:**

Mitochondrial dysfunction and mitochondrial DNA (mtDNA) damage in the retinal pigment epithelium (RPE) have been implicated in the pathogenesis of age-related macular degeneration (AMD). However, a deeper understanding is required to determine the contribution of mitochondrial dysfunction and impaired mitochondrial autophagy (mitophagy) to RPE damage and AMD pathobiology. In this study, we model the impact of a prototypical systemic mitochondrial defect, mitochondrial encephalomyopathy, lactic acidosis, and stroke-like episodes (MELAS), in RPE health and homeostasis as an *in vitro* model for impaired mitochondrial bioenergetics.

**Methods:**

We used induced pluripotent stem cells (iPSCs) derived from skin biopsies of MELAS patients (m.3243A > G tRNA leu mutation) with different levels of mtDNA heteroplasmy and differentiated them into RPE cells. Mitochondrial depletion of ARPE-19 cells (*p*^0^ cells) was also performed using 50 ng/mL ethidium bromide (EtBr) and 50 mg/ml uridine. Cell fusion of the human platelets with the *p*^0^ cells performed using polyethylene glycol (PEG)/suspension essential medium (SMEM) mixture to generate platelet/RPE “cybrids.” Confocal microscopy, FLowSight Imaging cytometry, and Seahorse XF Mito Stress test were used to analyze mitochondrial function. Western Blotting was used to analyze expression of autophagy and mitophagy proteins.

**Results:**

We found that MELAS iPSC-derived RPE cells exhibited key characteristics of native RPE. We observed heteroplasmy-dependent impairment of mitochondrial bioenergetics and reliance on glycolysis for generating energy in the MELAS iPSC-derived RPE. The degree of heteroplasmy was directly associated with increased activation of signal transducer and activator of transcription 3 (STAT3), reduced adenosine monophosphate-activated protein kinase α (AMPKα) activation, and decreased autophagic activity. In addition, impaired autophagy was associated with aberrant lysosomal function, and failure of mitochondrial recycling. The mitochondria-depleted *p*^0^ cells replicated the effects on autophagy impairment and aberrant STAT3/AMPKα signaling and showed reduced mitochondrial respiration, demonstrating phenotypic similarities between *p*^0^ and MELAS iPSC-derived RPE cells.

**Conclusions:**

Our studies demonstrate that the MELAS iPSC-derived disease models are powerful tools for dissecting the molecular mechanisms by which mitochondrial DNA alterations influence RPE function in aging and macular degeneration, and for testing novel therapeutics in patients harboring the MELAS genotype.

**Supplementary Information:**

The online version contains supplementary material available at 10.1186/s13287-022-02937-6.

## Introduction

Mitochondrial dysfunction is linked with a wide range of complex age-related ophthalmic diseases such as diabetic retinopathy, age-related macular degeneration (AMD), and glaucoma. Inherited mitochondrial DNA (mtDNA) mutations are associated with macular retinal pigment epithelium (RPE) disease in mitochondrial myopathies and retinal ganglion cell (RGC) loss in Leber hereditary optic neuropathy and other optic atrophy syndromes [1, 2]. Normal aging increases mitochondrial damage in the retina and RPE and extensive changes to the mitochondrial genome and increased reactive oxygen species (ROS) production have been associated with AMD pathogenesis [3]. AMD-related mtDNA damage has been found in RPE harvested from the macula and peripheral retina, but not from photoreceptors [4]. Thus, therapeutic strategies targeting RPE mitochondrial homeostasis may be relevant to prevent or reverse RPE degeneration in AMD.

The post-mitotic RPE is a pigmented cell monolayer between the retina and the choroid and is under considerable oxidative stress due to reactive oxygen species (ROS) generated in response to light, phagocytosis, and its high metabolic activity [5]. However, the molecular mechanisms that contribute to RPE damage and the development of AMD are not fully understood. Oxidative damage in the RPE leads to the accumulation of damaged cellular proteins and cellular organelles including mitochondria, which are eliminated by two major proteolytic systems involving the ubiquitin–proteasome and the lysosomal/autophagy pathways [6, 7]. Impaired mitochondrial autophagy (mitophagy) leads to reduced clearance of damaged mitochondria contributing to RPE degeneration [7, 8].

The mitochondrial genome is a target of ROS-induced mtDNA damage [7] and has a limited ability to repair mtDNA compared to nuclear DNA. Mutations in mtDNA genes can perturb mitochondrial bioenergetics and predispose cells to metabolic and degenerative diseases [9]. Healthy mammalian somatic cells are typically homoplasmic, (i.e., all copies of the mitochondrial DNA are identical). However, cells can contain both mutated and healthy mitochondria, termed heteroplasmy [10]. The threshold level of the heteroplasmic mtDNA is responsible for both the clinical manifestations of a mitochondrial disease and the degree of metabolic dysfunction. High levels of heteroplasmy for pathogenic mtDNA mutations are found not only in patients with mitochondrial disease, but are also seen with normal aging and increase with AMD severity [11]. The RPE is protected against ROS-induced mitochondrial damage by upregulating expression of antioxidants through endogenous pathways including peroxisome proliferator-activated receptor gamma coactivator I-alpha (PGC-1α[12]. Importantly, PGC-1*α* is a master regulator of mitochondrial biogenesis and also helps to maintain the functional and phenotypic state of RPE cells by supporting oxidative metabolism and autophagy-mediated repression of epithelial-mesenchymal transition (EMT) [13, 14]. PGC-1*α* is positively regulated by adenosine monophosphate-activated protein kinase (AMPK) *α* [15], which is an energy sensor and promotes autophagy and mitophagy [16]. Recently, PGC-1*α* gene was found to be repressed in RPE cells from AMD patients [17], suggesting that the interplay between AMPk*α*/PGC-1*α* signaling, and mitophagy may impact mechanisms of RPE energy homeostasis and play a role in the development or progression of AMD.

The cancer stem cell biomarker, Prominin-1 (Prom1) is upregulated in response to mitochondrial dysfunction suggesting that Prom1 expression is regulated by bioenergetic stresses [18]. Our previous studies showed that hypoxic stress and nutrient deprivation increases Prom1 expression in the human RPE [19], demonstrating a mechanistic relationship between environmental conditions (e.g., hypoxia), mitochondrial dysfunction, and Prom1 expression. Commonly, studies of RPE mitochondrial dysfunction use RPE lines depleted of mitochondrial DNA (Rho null, *p*^0^) by repeated passaging in ethidium bromide (EtBr) [20, 21] and other drugs [22]. However, the impact of mtDNA mutations and RPE mitochondrial turnover in real time cannot be modeled using this experimental strategy. Previously, the approach has been to use the cybrid (cytoplasmic hybrid) model, in which the mitochondria-depleted RPE cells are fused with mitochondria-rich human platelets [23, 24]. Cybrids have been shown to regain RPE cell morphology and molecular phenotype and have been useful for experimental modulation of severe mitochondrial dysfunction [25]. To better model the decline in mitochondrial function and heteroplasmy seen in aging and AMD, we generated mitochondria mutant RPE cells by differentiating induced pluripotent stem cells (iPSCs) from mitochondrial encephalomyopathy, lactic acidosis, and stroke-like episodes (MELAS) patients into RPE cells with different levels of mtDNA heteroplasmy. The phenotypic spectrum of the MELAS mutation includes retinal degeneration [26]. The focus of this study was to demonstrate that MELAS patient iPSC-derived RPE cells provide a platform for dissecting the impact of mitochondrial dysfunction and autophagy impairment on RPE function and establish a proof of concept for targeting the mitochondria in the treatment of AMD.

Our studies demonstrate the role of mitochondrial bioenergetics in RPE degeneration and identify the major signaling mechanisms regulating RPE autophagy and mitophagy associated with AMD pathobiology. Our data suggest that therapies aimed at activating RPE mitophagy may have applications in the prevention or treatment of AMD.

### Methods

The aims of the study were to generate cultures of differentiated RPE cells from MELAS iPSCs. These iPSC-derived cell lines were genotypically characterized with low or high mitochondrial heteroplasmy and were compared to control iPSC-derived RPE cells and to the standard mitochondrial model using Rho-null (*p*^0^) cells as novel disease model of defective cellular bioenergetics in the RPE.

## Reagents

Disposable cell culture ware was purchased commercially (Corning Glass Works, Corning, NY, USA). The ARPE-19 cell line was purchased from ATCC CRL-2302 (Manassas, VA, USA) and cultures were authenticated by ATCC’s STR profiling service. Cell culture medium was obtained from Lonza (Walkersville, MD, USA) and fetal bovine serum (FBS) was purchased from Atlanta Biologicals (Flowery Branch, GA, USA). Other materials purchased commercially were enhanced chemiluminescence (ECL) Western Blot detection system (PerkinElmer, Inc., Boston, MA, USA); LC3-I/II; p62/sequestosome; Cathepsin-D; PGC-1*α*; phospho-S6 Ribosomal protein Ser235/236; total-S6 Ribosomal protein; phospho-AMPK*α* Thr172, total-AMPK*α*; phospho-STAT3 pY705; total-STAT3; Oct4; Sox2; Nanog; TRA-1–60; and PINK1 antibodies (Cell Signaling Technology, Beverly, MA, USA); FG4592 and Erastin (Selleckchem, Houston, TX, USA); Carbonyl cyanide 3-chlorophenylhydrazone (CCCP), Uridine (Millipore, Sigma, St. Louis, MO, USA); BEST1; RPE65; CRALBP; and APOE antibodies (Santa Cruz Biotechnology, Dallas, TX, USA); Prom1/CD133 antibody (Aviva, San Diego, CA, USA); Bafilomycin A1 (EMD Millipore Corp., Billerica, MA, USA); PicoGreen, and MitoRed tracker (Invitrogen, Thermo Fisher Scientific, Waltham, MA, USA). All chemicals were of the highest purity commercially available.

## Cell and cybrid cultures

ARPE-19 cells were cultured in DMEM containing 10% FBS, 5% CO_2_, and passaged when 80% confluent, as described previously [19, 27]. Mitochondrial depletion was performed using 50 ng/ml ethidium bromide (EtBr) and 50 μg/ml uridine to obtain Rho-null (*p*^0^) cells, as described previously [21]. Transmitochondrial cybrids were created by fusing mtDNA-deficient ARPE-19 (*p*^0^) cell line with human platelets (commercially available from Cellero (Northridge, CA, USA). All experiments involving human cells were approved by the Vanderbilt University Institutional Biosafety Committee (VU IBC). Cell fusion was performed using a 1:1 (w/v) polyethylene glycol 1000 (PEG)/minimum essential medium for suspension cultures without calcium and glutamine (SMEM) mixture, followed by growth of fused cells in OptiMEM growth medium for up to 16 days, as described previously [23]. Passages were routinely tested for mycoplasma.

## Real-time quantitative PCR

TRIzol reagent (Thermo Fisher Scientific, Waltham, MA, USA) was used to extract total RNA from control ARPE-19 and EtBr-treated cells. Total RNA concentrations were quantified by measuring A260 and A280 using NanoDrop spectrophotometry, as described previously [19]. Total-RNA (1 μg) was reverse-transcribed to cDNA using a kit from Promega (Madison, WI, USA) and following manufacturer’s instructions. The cDNA was diluted 1:5 with DNase-free water. Real-time qPCR was performed using an Ariamx Real-Time PCR system (Agilent Technologies, Santa Clara, CA, USA) with 2.5 μl of the cDNA product in a 25 μl reaction mixture containing 1X SYBR® Green Master Mix (Applied Biosystems, Foster City, CA, USA) and 120 nM forward and reverse primers. The primers used for Cox4 (forward 5′- GCACTGAAGGAGAAGGAGAAG- 3″), reverse (5′- CCACAACCGTCTTCCACTC- 3′) sequences were used. The qPCR conditions were 50 ^°^C for 2 min, 95 °C for 10 min, followed by 40 cycles of 95 °C for 15 s and 60 °C for 1 min, as described previously. Each reaction was performed in triplicate.

## Human iPSC characterization and culture

Control and MELAS-iPSCs (with varying levels of mitochondrial heteroplasmy) were obtained from Timothy Nelson’s laboratory at Mayo Clinic (Rochester, MN, USA). Skin biopsies from non-mitochondrial disease patients (as controls) and MELAS patients (MitoC) with mutation in m.3243A > G in MT-TL1 (tRNA leu) were used to generate iPSCs as described earlier [28]. All skin fibroblasts were reprogrammed using CytoTune-iPS Sendai Reprogramming kits according to manufacturer’s instructions (Invitrogen, Thermo Fisher Scientific, Waltham, MA, USA). MELAS fibroblasts and derived iPSC cultures were supplemented with 50 μg/ml of uridine and maintained in mTeSR1 (STEMCELL Technologies, Vancouver, BC, Canada). The heteroplasmy levels of the mutation m.3243A > G in MELAS-iPSC clones were determined by polymerase chain reaction-restriction fragment length polymorphism (PCR–RFLP) and mtDNA next-generation sequencing analyses as described earlier [28]. Mito C-234 (low heteroplasmy) and Mito C-152 (high heteroplasmy) iPSCs were used for our studies.

## In vitro differentiation of iPSCs into RPE

Control and MELAS iPSCs were cultured in feeder-free, serum-free conditions in mTeSR1 medium to 80% confluency and subjected to in vitro RPE differentiation as described elsewhere [29]. Briefly, iPSC colonies from a single well of a 6-well plate were washed, treated with EDTA, and manually scraped for seeding into 4 wells of a 12-well plate coated with extracellular Matrigel basement membrane matrix (Corning, NY, USA; Cat # 356,237) following manufacturer recommendations. On day 1, the medium was changed using retinal differentiation medium (RDM) containing 10 mM nicotinamide (NIC) (Sigma, St Louis, MO, USA), 50 ng/ml noggin (R&D systems, Minneapolis, MN, USA), 10 ng/ml human Dickkopf WNT signaling pathway inhibitor 1 (DKK-1) (R&D systems, Minneapolis, MN, USA), and 10 ng/ml IGF-1 (R&D systems, Minneapolis, MN, USA) in Dulbecco’s modified essential medium/nutrient mixture F12 (DMEM/F12). On day 2, medium was changed using RDM containing 10 mM NIC, 5 ng/ml FGF-basic (PeproTech, Cranbury, NJ, USA), 10 ng/ml noggin, 10 ng/ml DKK-1, and 10 ng/ml IGF-1. On day 4, the medium was replaced using RDM containing 100 ng/ml activin A, 10 ng/ml DKK-1, and 10 ng/ml IGF-1. On day 6, the medium was replaced using RDM with 100 ng/ml activin A (PeproTech, Cranbury, NJ, USA) and 10 μm SU 5402 (FGF-receptor-specific tyrosine kinase inhibitor) (Santa Cruz Biotechnology, Dallas, TX, USA). On days 8, 10, and 12, the medium was changed using RDM with 100 ng/ml activin A, 10 μm SU 5402, and 3 μm CHIR99021 (glycogen synthase kinase 3, GSK-3*β *inhibitor) (Sigma, St. Louis, MO, USA). Confluent monolayers were washed with RDM (without growth factors), and non-pigmented cells were manually removed. Subsequently, the monolayers were treated with Trypsin-like dissociation enzyme (TDE) (TrypLE, Thermo Fisher, Waltham, MA, USA), and the cell/TDE suspension was centrifuged and resuspended in RPE-supporting medium (X-VIVO 10) (Lonza, Pittsburgh, PA, USA) containing ROCK inhibitor 10 μm Y-27632 (Tocris, Minneapolis, MN, USA). Cells were triturated to break into smaller clumps and seeded in 6-well plates coated with growth-factor reduced ECM with X-VIVO medium containing 10 μm Y-27632 (STEMCELL Technologies, Vancouver, BC, Canada). Culture medium was replaced every 3–4 days (without Y-27632) for 30 days. Cells were passaged at day 35 and re-seeded on growth-factor reduced Matrigel and allowed to mature for additional 30 days. The MELAS iPSC-234-RPE (with low mitochondrial heteroplasmy) and MELAS iPSC-152-RPE (with high mitochondrial heteroplasmy) cells required uridine supplementation in DMEM/F12 for growth.

## Primary human RPE cultures

Primary human RPE cells were isolated from postmortem de-identified donor eyes, as described earlier [19, 27]. The institutional review board at the University of Tennessee Health Science Center approved the use of human eyes from de-identified donors under an exempt protocol (46.104 Exempt research 4). All donor eyes were shipped to our laboratory within 24 h of enucleation. Globes were excised, anterior segment was removed, vitreous was extracted manually, and the retina was dissected free. The eyecup was washed three times with Dulbecco’s Modified Eagle’s Medium (DMEM), and 0.25% trypsin/EDTA, and loosened cells were aspirated, transferred to DMEM with FBS, and spun at 2000 g for 5 min. The cell pellet was resuspended in DMEM in 15% FBS and plated in poly-L-Lysine coated 12-well cell culture ware. Primary cultures within the first three to five passages were used for our studies. The specific studies performed using the RPE cells (approved, exempted) and reported in this paper were performed, while the authors were faculty and staff at the University of Tennessee Health Science Center.

## Transepithelial electrical resistance measurements

iPSC-RPE cells were cultured on growth-factor reduced Matrigel-coated transwell inserts (growth surface area, 0.33 cm^2^). After attachment of cells, culture media were replaced every 2–3 days. Transepithelial electrical resistance (TEER) was measured using an EVOM2 epithelial volt-ohmmeter (World Precision Instruments, Sarasota, FL, USA) with a chopstick STX2 electrode applied on the apical and basal sides of the RPE monolayers, as described earlier [30]. To obtain net TEER values of a target sample, the volt-ohmmeter output value of the blank transwell-insert lacking cells (with culture medium only) was subtracted from the sample output and multiplied by the surface area. TEER = (Sample resistance – blank resistance) x surface area, as described earlier [31]. Values were plotted as Ohms (Ω)/cm^2^.

## Mitochondrial defects

FLowSight Imaging cytometry methods [32] were used to quantify mitochondrial defects. Briefly, control and EtBr-treated ARPE-19 cells were rinsed with PBS and trypsinized. Cells were incubated with PicoGreen in culture medium at 37^0^C for 1 h followed by incubation with MitoTracker Red for 1 h and washed three times with 1X phosphate-buffered saline (PBS). Samples were analyzed with the FLowSight Imaging Flow Cytometer (EMD Biosciences/Millipore, Billerica, MA, USA), which produces dark field (side scatter), bright field (BF), and fluorescence images at ~ 20 × magnification. Compensation controls were obtained from single color-stained cells. The IDEAS software (Amnis Technology, EMD Millipore, Billerica, MA, USA) was used to separate single cells and cell doublets positive for PicoGreen^+^/MitoTracker Red^+^ cells. We used the Spot Counting Wizard in IDEAS to automatically quantify mitochondrial puncta formation. To correctly identify mitochondrial DNA, we manually identified cells expressing low (diffuse) and high (puncta) numbers of mitochondrial DNA puncta (red) and cellular DNA (green). A gradient histogram of the collected population was selected to choose the cells with better focus. The focused cells were gated and analyzed into a scatter plot of area versus aspect ratio. Single cells were plotted into a scatter plot of the cellular DNA (green intensity) versus the area of the merged (yellow intensity). Cells with mitochondrial puncta were defined as low (green) and high merged (yellow) contrast.

## Western Blotting

Cell lysates were prepared using mammalian protein extraction buffer (Cell Signaling Technology, Beverly, MA, USA) and a Halt protease/phosphatase inhibitor cocktail (Thermo Fisher Scientific, Waltham, MA, USA) followed by SDS-PAGE, as described previously [19, 27]. Proteins were transferred to Immobilon-PVDF 0.45 μm pore size membranes (Millipore, Bedford, MA, USA) and probed with primary antibodies overnight at 4 °C in Tris-buffered saline containing 0.1% Tween-20 and 5% nonfat dry milk (Biorad, Hercules, CA, USA). Membranes were subsequently incubated with horseradish peroxidase-conjugated secondary antibodies at room temperature for 1 h and the immune complexes were visualized by the ECL detected system (PerkinElmer, Waltham, MA) using the Azure c500 Imaging Biosystem (Dublin, CA, USA). Membranes were stripped and re-probed for actin as loading control. Representative Western Blots for three experiments are shown. Densitometric analysis of all Western Blots was performed using Image J software (developed by Wayne Rasband, National Institutes of Health, Bethesda, MD, USA; available at http://rsb.info.nih.gov/ij/index.html), as described earlier [19]. Blots used in the main figures are compliant with the digital image and integrity policies. The uncropped full-length blots are available as Additional file 1.

## Analysis of autophagy flux

Control, MELAS iPSC-234-RPE with low mitochondrial heteroplasmy (het^low^), and MELAS iPSC-152-RPE with high mitochondrial heteroplasmy (het^high^) were treated with 20 μm carbonyl cyanide m-chlorophenylhydrazone (CCCP) for 3 h followed by an additional treatment of bafilomycin for 3 h in the presence of CCCP, as described earlier [33, 34]. Cells were lysed, and immunodetection of LC3 was performed as described above. To quantify autophagic synthesis, we calculated the LC3-II ratio between the conditions where we induced autophagy by CCCP treatment and blocked lysosomal degradation by bafilomycin treatment divided by the condition where the cells were treated with bafilomycin alone. In addition, we quantified autophagic degradation by calculating the relation where we induced autophagy by CCCP and blocked lysosomal degradation by bafilomycin divided by the condition where autophagy was induced by CCCP (Fig. -6B), as described earlier [34].

## Seahorse analysis of mitochondrial function

Using a Seahorse XP/XF96 analyzer (Agilent, Santa Clara, CA, USA), we measured oxygen consumption rate (OCR), an indicator of mitochondrial respiration; and extracellular acidification rate (ECAR), an indicator of aerobic glycolysis, as described previously [22]. Control ARPE-19 cells and cells treated with 50 ng/ml EtBr for 7 days, were trypsinized and seeded into XFp cell culture Microplates (Agilent, Santa Clara, CA, USA) at 7000 cells/well one day before the assay. Additionally, MELAS iPSC-derived and control iPSC-derived RPE cells were seeded into XFp microplates for assessment of mitochondrial function, as described previously [12]. The following day, the culture medium was changed to XF Assay Medium (Agilent, Santa Clara, CA, USA) containing 1 mM pyruvate, 2 mM glutamine, and 10 mM glucose. Mitochondrial stress test was performed after injecting oligomycin (2 μm/well), FCCP (2 μm/well), and rotenone/antimycin A (0.52 μm/well). After addition of each reagent, three separate measurements were collected. OCR and ECAR values were normalized to the total number of cells per well. All values were calculated as pmol/min/cell, following manufacturer’s instructions.

## Statistical analysis

All data were analyzed by GraphPad Prism 9 program (GraphPad Software Inc., San Diego, CA). Data are expressed as mean ± SE. Experiments were repeated three times, with triplicate samples for each. An unpaired 2-tailed Student’s t test and Bonferroni post hoc testing were used to assess statistical significance. Unless otherwise stated, values of *P < 0.05; **P < 0.01; ***P < 0.001, and ****P < 0.0001 were considered significant.

## Results

### Mitochondrial DNA depletion by EtBr and rescue by fusion cybrids in ARPE-19 cells

**Fig. 1:**
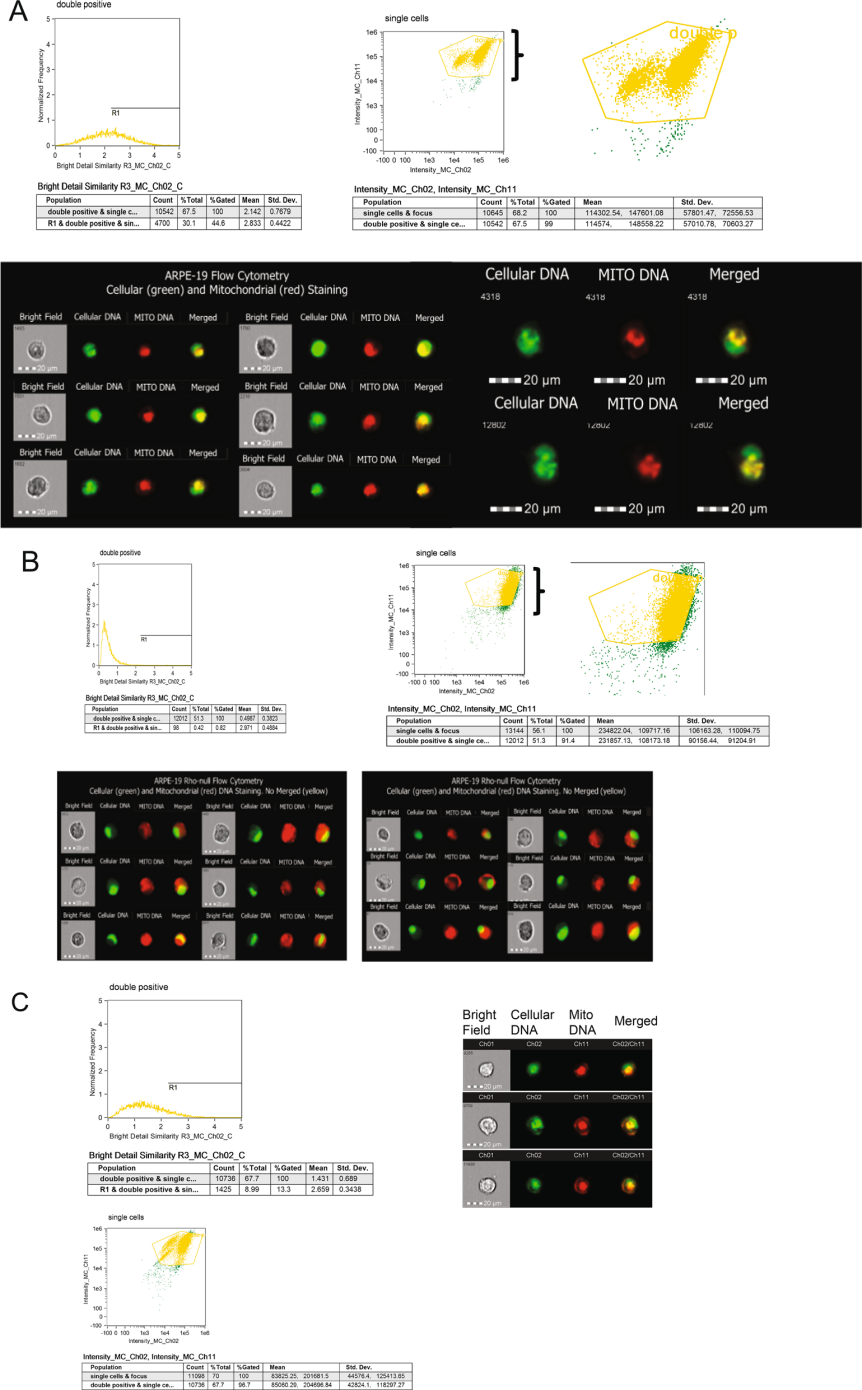
FLowSight cytometry of control and *p*^0^ cells and rescue of mitochondrial function by cybrids. **A** Control and **B** EtBr-treated *p*^0^ cells were stained with PicoGreen and MitoTracker Red dyes. Single cells were analyzed by FLowSight cytometry, and the IDEAS software was used to analyze cellular (green), mitochondrial (red) DNA staining, and merged (yellow). **C** Cybrids were analyzed by FLowSight cytometry for the presence of cellular (green) and mitochondrial DNA (red)

To determine whether we can detect and monitor progressive mitochondria depletion, we treated proliferating RPE cells (ARPE-19 cells) with a low dose of ethidium bromide (EtBr, 50 ng/ml) for up to 16 days. PicoGreen and MitoTracker Red fluorescent dyes were used to visualize mtDNA in living cells. [35] We used fluorescent PicoGreen to detect cellular double-stranded DNA (dsDNA), and cell-permeable MitoTracker fluorescent Red CMXROs to detect mitochondrial DNA in live cells. We used multispectral imaging flow cytometry, which permits multiparametric images of single cells (at rates up to 1000 cells/sec) [19] to characterize control-RPE cells, *p*^0^cells, and cybrid-rescued RPE cells. Untreated cells showed strong PicoGreen staining of nuclear DNA and red fluorescence showing the presence of mitochondria. We selected a gradient histogram of the collected population to choose cells, which were gated and analyzed into a scatter plot of Area versus Aspect ratio. Single cells were plotted into a scatter plot of the cellular DNA (green) versus the merged area (yellow). Double positive merged (yellow) images were defined as cells with high cellular and mitochondrial DNA (Fig. 1A). Control cells showed 30.1% double positive cells with 44.6% gated, which decreased to 0.4% with 0.82% gated in EtBr treated for 16 days *p*^0^ cells, showing mitochondrial depletion (Fig. 1B). To demonstrate that *p*^0^ cells can be rescued, we performed cybrid fusion of *p*^0^ cells with human platelets, which contain an abundance of mitochondria. Platelet fusion (cybrid formation) restored mitochondrial staining in mitochondrial depleted cells as evidenced by 8.99% double positive cells with 13.3% gated (Fig. 1C). We analyzed expression of mitochondrial proteins cytochrome C oxidase subunit 4 (COX4), voltage-dependent anion channel 1 (VDAC1), and prohibitin 1 (PHB1) through the process of mitochondrial depletion and cybrid rescue (Additional file 1: Fig. S1). The COX4, PHB1, and VDAC protein levels were barely detectable after 10 days of EtBr treatment in *p*^0^ cells (Additional Fig. S1A left panel). We observed a similar decrease in Cox4 and VDAC protein levels and Cox4 mRNA after 9 days of EtBr treatment in *p*^0^ cells. Platelet fusion robustly restored Cox4 mRNA levels and expression of both proteins (Additional file 1: Fig. S1A, right panel), demonstrating that cybrid fusion is capable of rescuing mitochondrial function in RPE cells.

**Fig. 2:**
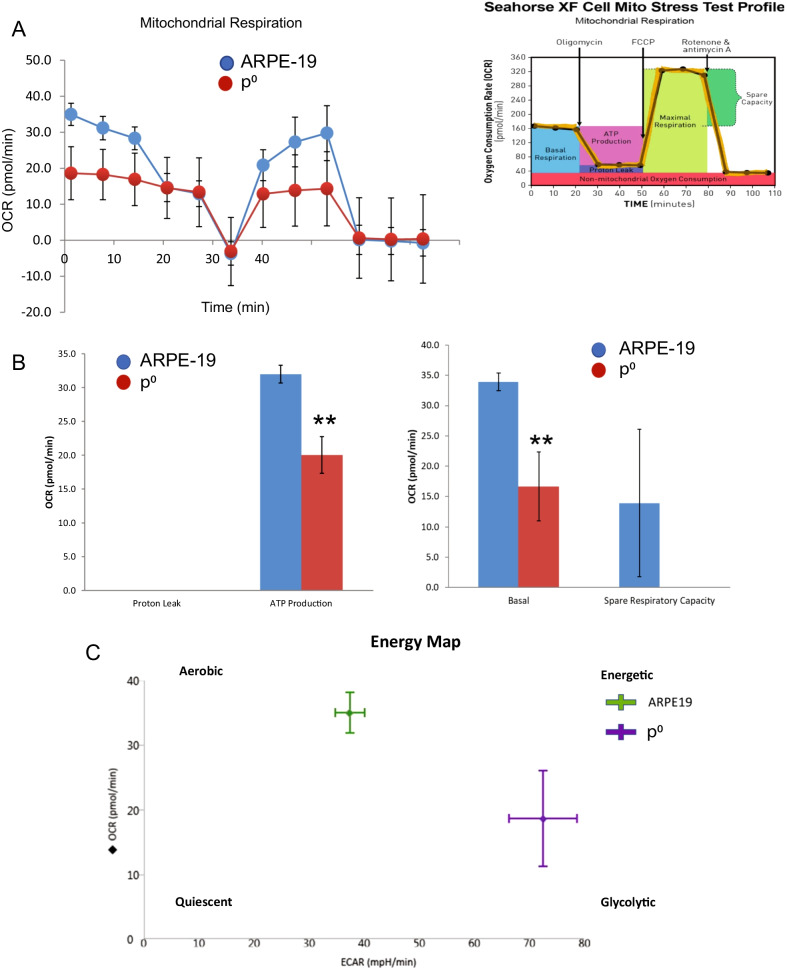
Mitochondrial energetics of *p*^0^ cells.** A** Mitochondrial respiration and OCR in control and EtBr-treated (*p*^0^) ARPE-19 cells. **B** ATP production, basal and spare respiratory capacity in control and *p*^0^ cells. Control versus *p*^0^ cells; *p* < 0.01. **C** Energy map showing changes in OCR and ECAR

Since mitochondrial dysfunction creates an imbalance between aerobic glycolysis and oxidative phosphorylation [22], we used the Seahorse XF mitochondrial stress test to examine mitochondrial energy dynamics in studies of *p*^0^ cells. We found that p^0^ cells have significantly lower oxygen consumption rate (OCR) and decreased ATP production (Fig. 2A-B). This was accompanied by an increased extracellular acidification rate (ECAR), demonstrating a shift toward aerobic glycolysis (Fig. 2C). As expected, *p*^0^ cells are unable to meet their energy requirements via oxidative phosphorylation.

## Molecular mechanisms associated with mitochondrial dysfunction in ***p***^0^ ARPE-19 cells

**Fig. 3:**
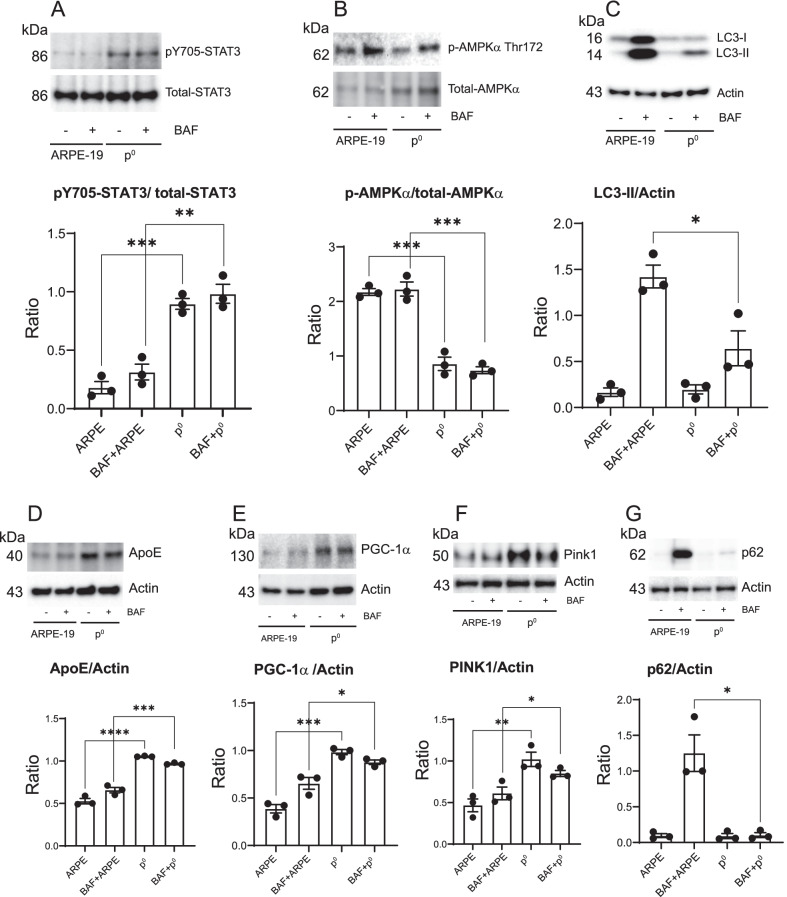
Autophagy and mitophagy signaling in control and *p*^0^ cells. Control and *p*^0^ ARPE-19 cells were treated with or without bafilomycin 100 nM for 3 h. Cell lysates were analyzed by Western Blotting using indicated antibodies. Cropped representative blots from *n* = 2 experiments. Quantification of **A** pYSTAT3/total-STAT3 ratio. **B** p-AMPKα/total-AMPKα ratio. **C** LC3-II/actin ratio. **D** ApoE/actin ratio. **E** PGC-1α/actin ratio. **F** PINK1/actin ratio. **G** p62/actin ratio. Full-length gels are presented in Additional file 1

To provide further evidence of a direct role of mitochondrial health in regulating autophagic and mitophagic activities in ARPE-19 cells, control and *p*^0^ cells were analyzed for the expression of key regulators of autophagy and RPE homeostasis. STAT3 localizes in mitochondria [36], binds with complex I of the electron transport chain [37], impacts ATP production [38], and regulates autophagy [39]. We investigated STAT3 activation in *p*^0^ cells. We observed significantly higher STAT3 activation (ratio of pY705-STAT3 to total-STAT3) in *p*^0^ cells demonstrating that mitochondrial (and thus energy) depletion induces STAT3 activation (Fig. 3A). Activation of AMPK*α*, a major regulator of autophagy in RPE cells (measured as the ratio of phospho-AMPK*α* Thr 172 to total-AMPKα, was significantly reduced in *p*^0^ cells compared to control cells (Fig. 3B). Treatment of *p*^0^ cells with bafilomycin did not alter STAT3 and AMPK*α* activation suggesting that these upstream signaling molecules are not regulated by inhibition of autophagy flux using bafilomycin. We investigated the impact of mitochondria depletion on autophagy flux by monitoring the conversion of soluble cytoplasmic LC3-I to the lipid-bound LC3-II form in the presence and absence of bafilomycin. The expected increase in LC3-II/actin ratio was observed in bafilomycin-treated control cells. However, the LC3-II/actin ratio was significantly lower in *p*^0^ cells, suggesting that mitochondrial dysfunction reduces autophagy flux and thus negatively impacts RPE homeostasis (Fig. 3C). Since impaired autophagy and oxidative stress are linked with the upregulation of AMD-associated genes such as apolipoprotein E (APOE) [40], we quantified APOE expression and found that it is significantly increased in *p*^0^ cells (Fig. 3D) suggesting that reduced autophagy flux in cells with loss of mitochondrial function correlates with increased APOE accumulation.

RPE autophagy flux depends on PGC-1*α* and its loss promotes retinal degeneration [14]. Mitochondrial depletion significantly increased PGC-1*α* expression suggesting that PGC-1*α* compensates for the loss of mitochondrial function in *p*^0^ cells by promoting mitochondrial biogenesis (Fig. 3E). This is consistent with earlier studies that demonstrated a similar role of PGC-1*α *in compensating for mitochondrial respiratory chain defects [41]. We observed that PGC-1*α *levels were unaltered in *p*^0^ cells treated with bafilomycin suggesting that inhibition of autophagy in *p*^0^ cells has no effect on PGC-1*α* expression. Mitochondrial dysfunction stabilizes a PTEN-induced serine/threonine kinase 1 (PINK1), by recruiting Parkin E3 ubiquitin ligase for mitophagy [42]. We analyzed PINK1 expression in *p*^0^ cells. Our results show that mitochondrial depletion significantly upregulates PINK1 expression (Fig. 3F), to remove damaged mitochondria by upregulation of PINK1-dependent mitophagy.

Since p62/sequestosome 1 (SQSTM1) functions as a sensor of mitophagy [43] and promotes mitochondrial ubiquitination [44], we analyzed p62 expression in *p*^0^ cells. As expected, bafilomycin significantly increased p62 levels in control-RPE cells demonstrating inhibition of its degradation, showing inhibition of autophagy. However, the expected increase in p62 levels in mitochondria-depleted cells was not seen (Fig. 3G). We hypothesize that this could be due to elevated p62 ubiquitination during mitophagy [44] or redox sensing by p62 [45] in response to loss of mitochondrial function, or both.

## Phenotype modeling of the MELAS m.3243A > G tRNA leu mutation in the RPE

Although EtBr-mediated mitochondria depletion is a powerful and easy method to deplete mtDNA, [46], studies show that EtBr can damage nuclear DNA and accumulates in the mitochondrial matrix leading to changes in cell morphology and function [46–48]. Thus, *p*^0^ cells are not an optimal model for mitochondrial bioenergetics in the RPE. Our goal was to model RPE mitochondrial disorders, identify functional deficiencies, and characterize clinically relevant disease phenotypes.

**Fig. 4:**
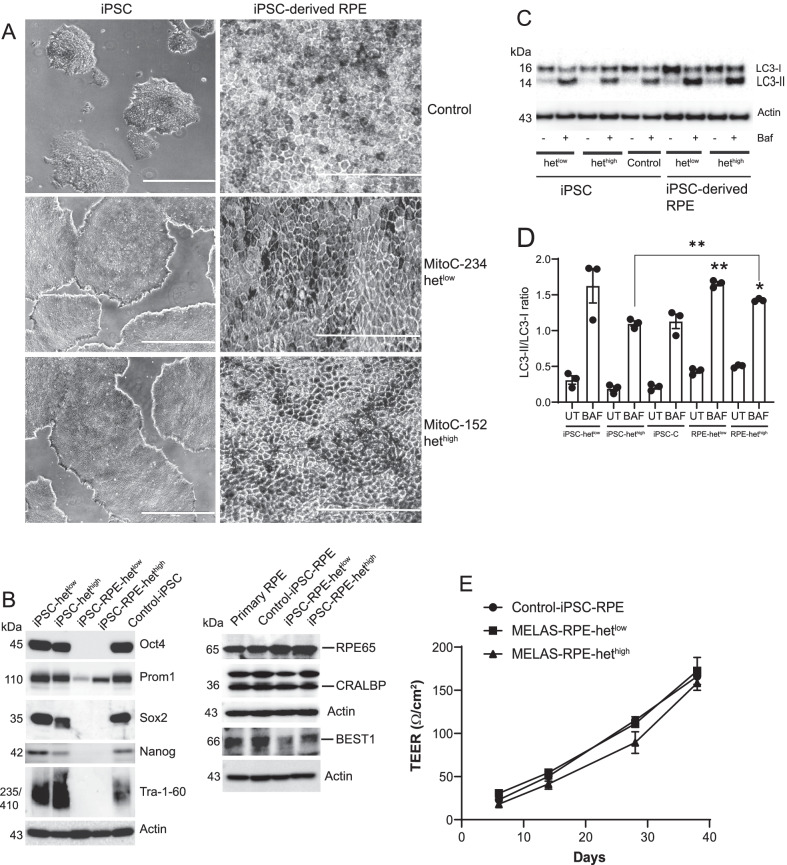
Generation and characterization of RPE with low and high mitochondrial heteroplasmy from MELAS-iPSCs. **A** MELAS het^high^ and het^low^ iPSCs (scale bar 40 μm) were differentiated to RPE cells (scale bar 200 μm). **B** Expression of pluripotency markers in iPSCs and iPS-derived RPE cells. Expression of RPE-specific proteins in iPSC-derived RPE and primary human RPE cells. Cropped representative blots are presented. **C** Autophagy flux in iPSCs and iPSC-derived RPE cells. Control, MELAS iPSCs, and iPSC-derived RPE cells were treated with or without bafilomycin (100 nM) for 3 h, and cell lysates were analyzed for LC3 by Western Blotting. A cropped representative LC3 blot is presented. **D** Densitometric analysis of LC3-II/LC3-I ratio. **E** TEER values from control, high, and low heteroplasmy iPSC-RPE cultured in transwell inserts. Full-length gels are presented in Additional file 1

To model RPE cells possessing mtDNA mutations, we obtained control iPSCs and MELAS -het^low^ and het^high^ iPSCs and differentiated them into RPE cells. The iPSCs formed colonies in culture (Fig. 4A) and expressed octamer-binding transcription factor 4 (Oct4), sex-determining region Y-box transcription factor 2 (Sox2), transcription factor Nanog, and Tra-1–60, confirming the presence of key pluripotency markers [49, 50] (Fig. 4B). The iPSCs were differentiated into RPE cells using published protocols [28, 51]. The iPSC-derived het^high^ and het^low^ RPE cells did not express Oct4, Sox2, Nanog, and Tra-1–60 (Fig. 4B) confirming differentiation. Given that CD133/Prom1 is highly expressed in stem and progenitor cells [52], we examined Prom1 expression in control and MELAS iPSCs. Our data show strong Prom1 expression in control and MELAS iPSCs. The het^low^ RPE cells showed low levels of Prom1 expression, which is consistent with our previous studies showing Prom1 expression in primary RPE cells from human donors [19]. However, the het^high^ RPE cells showed significantly increased Prom1 expression suggesting that Prom1 is upregulated in cells with high heteroplasmy. Generation of RPE cells from iPSCs was further shown by light microscopy, identifying typical epithelial cobblestone morphology and the presence of progressive pigmentation with extended culture on transwell plates. (Fig. 4A). To confirm RPE differentiation, we measured protein expression of RPE markers in the iPSC-derived cells. By onset of pigmentation, RPE-specific proteins [53] such as cellular retinaldehyde-binding protein (CRALBP), Bestrophin-1 (BEST1), and retinoid isomerohydrolase RPE65 were expressed. The expression levels of these proteins were similar across iPSC-derived RPE cells. The degree of mitochondrial heteroplasmy had no effect on the expression of RPE-specific proteins (Fig. 4B) in iPSC-derived RPE cells.

We measured autophagy flux in iPSCs and iPSC-derived RPE cells (Fig. 4C, D). These studies show higher autophagy flux in het^low^ iPSCs, but similar autophagy flux in control and het^high^ iPSCs (Fig. 4C, D). We compared autophagy flux between differentiated het^low^ RPE and undifferentiated het^low^-iPS cells, and there was no statistical variation. However, in iPSC-derived het^high^ RPE cells we found that differentiated RPE cells demonstrated enhanced autophagic activity compared to the parental and control iPSCs (Fig. 4C, D). This is consistent with the increase in autophagy in iPSC-derived RPE cells following stem cell differentiation [54].

Transepithelial electrical resistance (TEER) across the RPE layer is a measure of tight junction formation. We measured the TEER values of iPSC-RPE cells cultured on ECM-coated transwell inserts. Recordings at indicated time periods demonstrate a steady increase in the TEER by 38 days of culture (Fig. 4E). The TEER values are consistent with the reported values of iPSC-derived human RPE cells [55]. We found that both control iPSC-RPE cells (control-RPE) and MELAS -het^low^ RPE had similar levels of TEER, whereas MELAS het^high^ RPE showed low TEER values throughout the entire time period (Fig. 4E). We posit that this is an experimental artifact and is in part due to requirement for uridine supplementation in the culture medium. Both het^low^ and het^high^ cells required uridine containing media, and its removal prevented growth and reduced TEER values in het^high^ cells (data not shown). We believe that inadequate energy levels in het^high^ cells affect proliferation and junction formation, and uridine supplementation [26, 28] was required to mitigate these effects. We predict that control and MELAS iPSC-RPE cells are capable of establishing an appropriate epithelial barrier in vitro when supplemented [26], but the formation of tight junctions and TEER may be reduced in situ.

## Autophagy signaling in MELAS iPSC-RPE cells

**Fig. 5:**
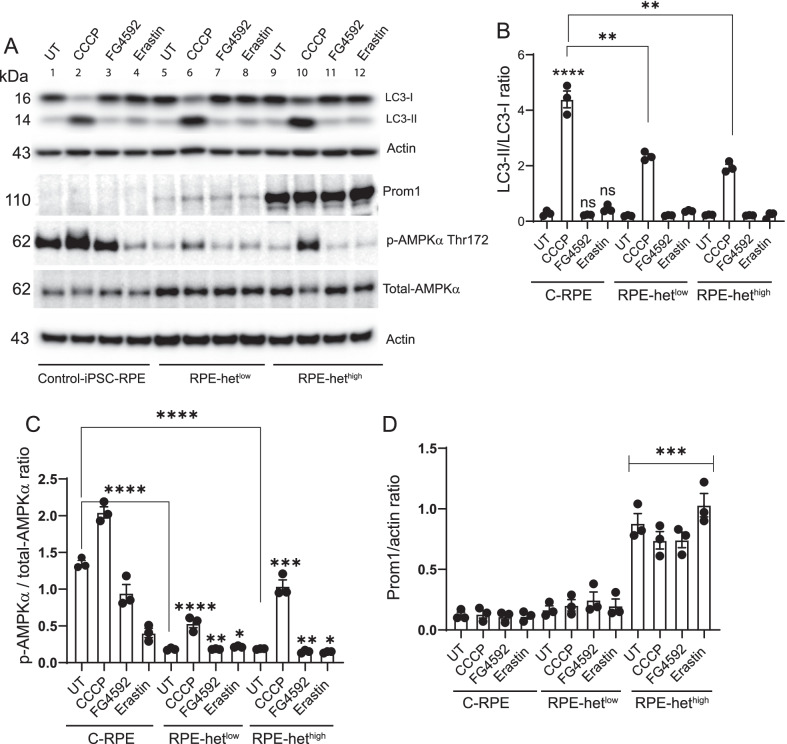
Upregulation of Prom1 expression and inhibition of AMPKα signaling in MELAS iPSC-derived RPE cells.** A** Differentiated control iPSC-RPE, iPSC-RPE het^low^ and iPSC-RPE het^high^ cells were treated with 20 μm CCCP for 6 h, 20 μm FG4592 overnight, and 10 μm Erastin for 6 h. Cell lysates were analyzed by Western Blotting using indicated antibodies, and cropped representative blots are presented. **B** Densitometric analysis of LC3-II/LC3-I ratio. **C** phospho-AMPKα/total-AMPKα ratio. **D** Densitometric analysis of Prom1/actin ratio. Full-length gels are presented in Additional file 1

To investigate the functional impact of mitochondrial heteroplasmy on MELAS-RPE in more depth, we treated cells with the respiratory chain uncoupler carbonyl cyanide m-chlorophenylhydrazone (CCCP, which triggers loss of mitochondrial membrane potential), hypoxia-inducible factor (HIF)-prolyl hydroxylase inhibitor FG4592 (used to mimic hypoxia in vitro), and the ferroptosis inducer Erastin, (which inhibits VDAC1, a multifunctional mitochondrial protein). Western Blot analysis revealed a significant increase in autophagy in control-RPE cells treated with 20 μm CCCP, but no difference in response to 20 μm FG4592 and 10 μm Erastin treatment (Fig. 5A, B). We observed significantly diminished relative autophagy induction in MELAS iPSC-RPE cells with het^low^ and het^high^ after treatment with CCCP (Fig. 5A, B). Both FG4592 and Erastin failed to induce autophagy strongly suggesting that autophagic activity is reduced in MELAS-RPE cells. To explore this, we examined the levels of AMPK*α* activation and Prom1 expression. It has been shown that Prom1 [19] and AMPK*α* activation [16] upregulate autophagy. Western Blot analysis showed a significant decrease in basal AMPK*α* activity in MELAS-RPE with het^low^ and het^high^ compared to control-RPE (Fig. 5A, C). Treatment of control-RPE with CCCP significantly increased AMPK*α* activation, which contributed to autophagy induction. The MELAS-RPE cells harboring different levels of mitochondrial heteroplasmy exhibited significantly less AMPK*α* activation after treatment with CCCP compared to control-RPE cells (Fig. 5A, C). Both FG4592 and Erastin reduced basal AMPK*α* activation in control-RPE but had no significant effect in MELAS-RPE cells. The level of AMPK*α* activation in response to FG4592 and Erastin was significantly lower in het^low^ and het^high^ cells compared to controls. These observations demonstrate that control-RPE cells respond to hypoxic stress and VDAC inhibition by reducing AMPK*α* activation. However, MELAS-RPE cells have low basal AMPK*α *and, therefore, FG4592 and Erastin are ineffective in downregulating its activation. Consequently, these inhibitors failed to stimulate autophagy in MELAS-RPE cells.

To assess the connection between Prom1 and autophagy regulation in iPSC-RPE cells, we measured Prom1 expression. Prom1 expression was barely detectable in control-RPE and increased in MELAS-RPE with het^low^. However, Prom1 expression significantly increased in MELAS-RPE with het^high^ in the presence and absence of CCCP, FG4592, and Erastin (Fig. 5A, D). This increase in Prom1 expression in MELAS-RPE suggests that Prom1 may compensate for high mitochondrial heteroplasmy by maintaining basal autophagic activity.

## Impairment of autophagy and imbalance of mitophagy-associated proteins in MELAS iPSC-derived-RPE cells

**Fig. 6:**
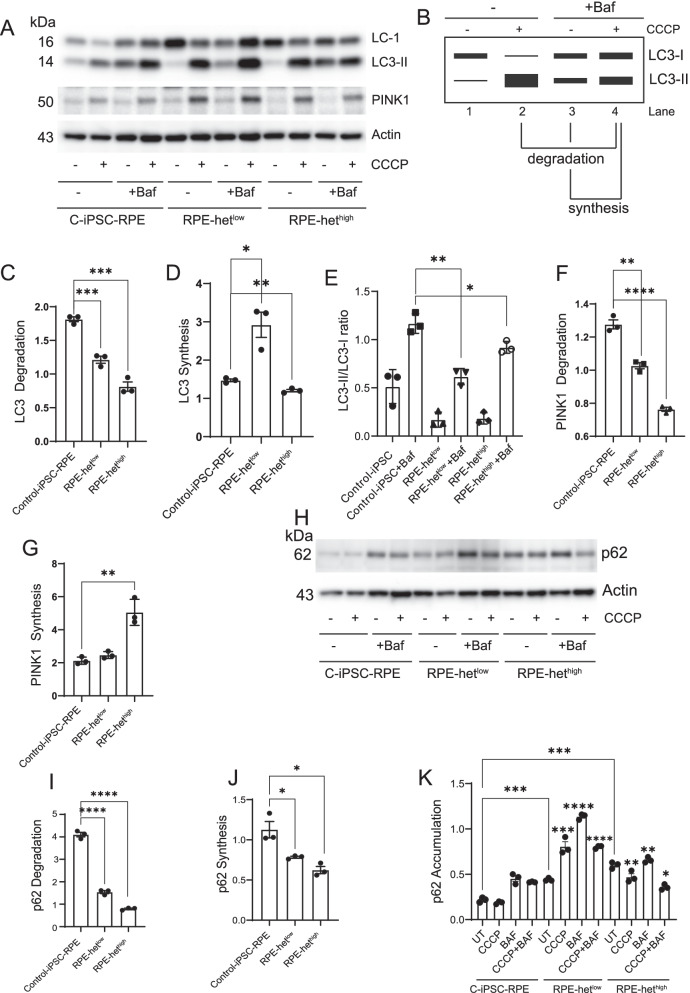
Impaired autophagy flux and defective degradation of LC3, p62, and PINK1 in MELAS iPSC-derived-RPE cells. **A** Cropped representative Western Blotting of LC3-I and LC3-II for the study of autophagy flux in control IPSC-RPE, iPSC-RPE-het^low^, and iPSC-RPE-het^high^ treated with or without CCCP, 20 μm for 3 h in the presence or absence of bafilomycin, 100 nM for 3 h. **B** Schematic for quantifying autophagy flux. **C** Quantification of LC3 degradation. **D** LC3 synthesis. (**E**) LC3-II/LC3-I ratio. **F** PINK1 degradation. **G** PINK1 synthesis. **H** Cropped representative Western Blotting of p62 and actin under identical experimental conditions. **I** Quantification of p62 degradation. **J** p62 synthesis. **K** p62 accumulation. Full-length gels are presented in Additional file 1

To confirm that inhibition of autophagy flux in MELAS-RPE cells corresponds to the level of mtDNA heteroplasmy, we treated cells with the mitochondrial uncoupler CCCP (20 μm), which triggers loss of mitochondrial potential. Cells were treated with CCCP for 3 h followed by an additional treatment with bafilomycin A1 for 3 h in the presence and absence of CCCP to inhibit autophagy degradation (Fig. 6A). Autophagy flux was quantified using the schematic (Fig. 6B) and described in Methods. Quantification of autophagy (LC3) synthesis was calculated as the ratio between the values of the cells treated with CCCP and bafilomycin relative to treatment with bafilomycin alone. Quantification of autophagic degradation was calculated as the ratio between the values of the cells treated with CCCP and bafilomycin and the ones with CCCP alone according to current autophagy interpretation guidelines [34]. We saw reduced autophagy flux where the autophagosome (LC3-II) degradation was significantly reduced in MELAS-RPE cells (Fig. 6C). The inhibition of LC3-II degradation directly correlated with the level of heteroplasmy, suggesting that this may be a modifiable phenotype. These observations were associated with a decrease in LC3-II synthesis in MELAS-RPE with het^high^ but a significant increase in MELAS-RPE with het^low^ (Fig. 6D), suggesting that a low burden of heteroplasmic mtDNA permits the upregulation of autophagosome synthesis for removal of dysfunctional mitochondria. However, autophagy flux (LC3-II/LC3-I ratio in the presence of bafilomycin) was significantly downregulated in het^low^ cells (Fig. 6E), which suggests that the inhibition of LC3 degradation pathway leads to reduction of autophagy flux (Fig. 6C). In het^high^ MELAS-RPE cells, both LC3 synthesis and its degradation were significantly inhibited (Fig. 6C, D), indicating that reduced autophagy flux cannot cope with the high burden of damaged mitochondria and leads to accumulation of dysfunctional mitochondria. Given that autophagy provides substrates for mitochondrial metabolism to avert energy crisis [56] and autophagic removal of damaged mitochondria is important for mitochondrial quality control, we propose that impaired autophagy in MELAS-RPE cells alters mitochondrial homeostasis leading to bioenergetic dysfunction.

We investigated whether mitochondrial recycling is impacted by the presence of mtDNA mutation. We observed a decrease in PINK1 degradation in MELAS-RPE, which correlated with the level of heteroplasmy (Fig. 6F). Furthermore, PINK1 synthesis was not significantly altered in het^low^ cells, but was significantly increased in het^high^ cells (Fig. 6G). These observations confirm previous findings that severe impairment of mitochondrial function increases PINK1 synthesis to upregulate mitophagy [57].

To examine the defect in autophagosome degradation, we examined p62 turnover. The basal levels of p62 are low in control-RPE cells and bafilomycin treatment in the presence and absence of CCCP increased p62 accumulation. The het^low^ and het^high^ MELAS-RPE cells exhibited significantly lower p62 degradation and p62 synthesis (Fig. 6I-J). Under basal conditions, p62 accumulation significantly increased in het^low^ and het^high^ MELAS-RPE, which further increased in the presence of bafilomycin (Fig. 6K) demonstrating inhibition of autophagy. CCCP treatment also significantly increased p62 accumulation in both het^low^ and het^high^ MELAS-RPE compared to control cells, confirming impairment of autophagy induction. Similarly, inhibition of LC3-II degradation and accumulation of p62 in MELAS iPSC-RPE cells suggest impairment of lysosomal function.

**Fig. 7:**
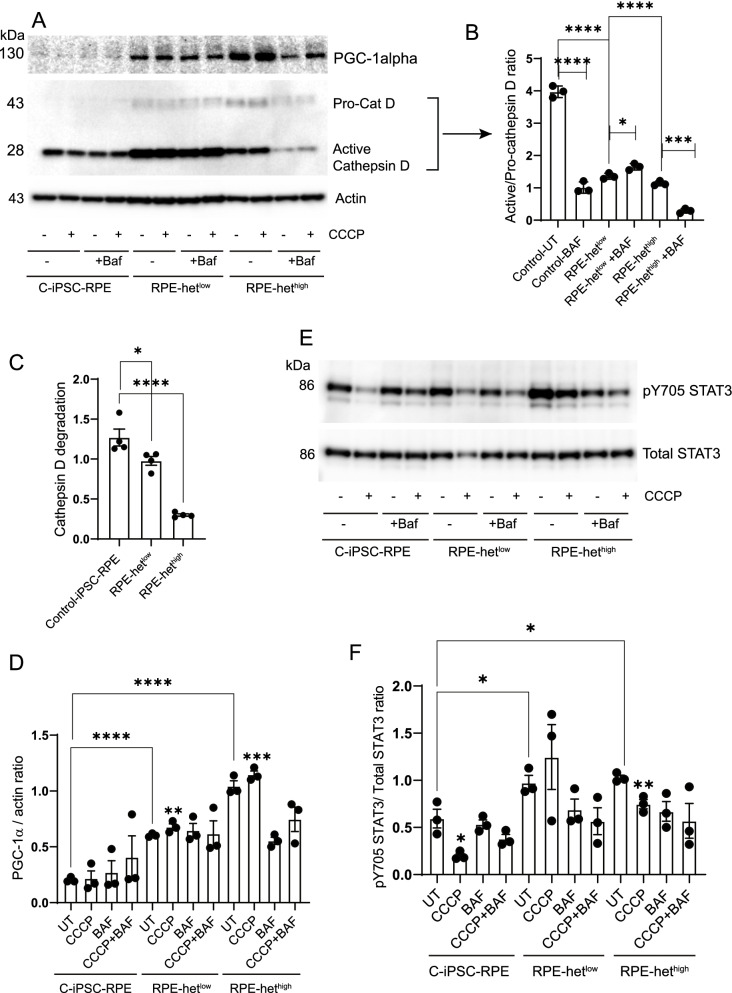
Activation of STAT3 correlates with inhibition of lysosomal cathepsin D and increased expression of PGC-1α in MELAS-RPE cells.** A** control iPSC-RPE, iPSC-RPE-het^low^, and iPSC-RPE-het^high^ cells were treated with 20 μm CCCP in the presence and absence of bafilomycin. Cell lysates were analyzed by Western Blotting using specified antibodies and cropped representative blots are presented. **B** Quantification of active/pro-cathepsin D ratio. **C** Quantification of cathepsin D degradation. **D** Quantification of PGC-1α/actin ratio.** E** Western blotting with pY705 STAT3 and total STAT3 antibodies. **E** Quantification of pY705 STAT3/total-STAT3 ratio. Full-length gels are presented in Additional file 1

To examine lysosomal dysfunction, we studied the processing of a major RPE proteolytic enzyme, cathepsin D, that is dependent upon lysosomal function [58]. Although basal levels of pro-cathepsin D were higher in MELAS-RPE, the activation ratio of active/pro-cathepsin D showed significantly lower cathepsin D activity in MELAS het^high^ and het^low^ cells compared to control-RPE, suggesting impaired lysosomal function in the MELAS-RPE (Fig. 7A-B). Bafilomycin significantly decreased cathepsin D activation in control-RPE and het^high^ cells but not in het^low^ cells likely because bafilomycin raises lysosomal pH [59] causing cathepsin D degradation (Fig. 7B). These observations suggest differential regulation of cathepsin D processing in cells with varying levels of heteroplasmy and imply lysosomal heterogeneity in RPE with mitochondrial dysfunction. Alteration of the acidic hub in the lysosome inhibits the physiological function of cathepsin D and disrupts its degradative capacity [60]. Consistent with this concept, cathepsin D degradation was significantly impaired in MELAS-RPE, more robustly in het^high^ cells (Fig. 7C), suggesting that damaged mitochondria cannot be adequately recycled. These observations suggest that stimulating cathepsin D activation in MELAS-RPE may be an approach to increase lysosomal function and promote mitophagy.

Since PGC-1*α *upregulates expression of antioxidants that protect against ROS-induced mitochondrial damage [12], we investigated PGC-1*α *expression in MELAS-RPE cells. We found that MELAS heteroplasmy significantly increased PGC-1*α* expression compared to control-RPE (Fig. 7A, D) suggesting that enhancement of PGC-1*α* may alleviate mitochondrial dysfunction by promoting the mitochondrial antioxidant defense in RPE cells. Similar to our earlier observations (Fig. 6), we found that the extent of PGC-1*α* upregulation is dependent on the level of heteroplasmy. As expected, CCCP significantly increased PGC-1*α* expression in both het^high^ and het^low^ cells demonstrating that loss of mitochondrial membrane potential upregulates PGC-1*α* These observations correlate with our earlier observations (Fig. 3) showing upregulation of PGC-1*α* expression in *p*^0^ ARPE-19 cells. We propose that PGC-1*α* is an important contributor to maintaining RPE mitochondrial health.

Since our data with *p*^0^ ARPE-19 cells showed that STAT3 activation correlates with autophagy inhibition, we compared STAT3 activation in MELAS-RPE. In both het^low^ and het^high^ cells, basal STAT3 activation was significantly higher than control-RPE cells (Fig. 7E-F), which correlated with reduced autophagy flux in MELAS iPSC-derived RPE cells (Fig. 6C, E) suggesting that STAT3 represses autophagy in these cells. The mitochondrial respiratory chain uncoupler CCCP significantly inhibited STAT3 activation in control and het^high^, but the changes seen in het^low^ cells were not statistically significant (Fig. 7E-F). Our earlier observations showed that CCCP significantly activated autophagy in control-RPE cells, but the level of autophagy induction was reduced in MELAS-RPE cells (Fig. 5A-B). Bafilomycin plus CCCP did not inhibit STAT3 activation in MELAS-RPE cells suggesting that impaired autophagy in these cells may be associated with STAT3 activation.

**Fig. 8:**
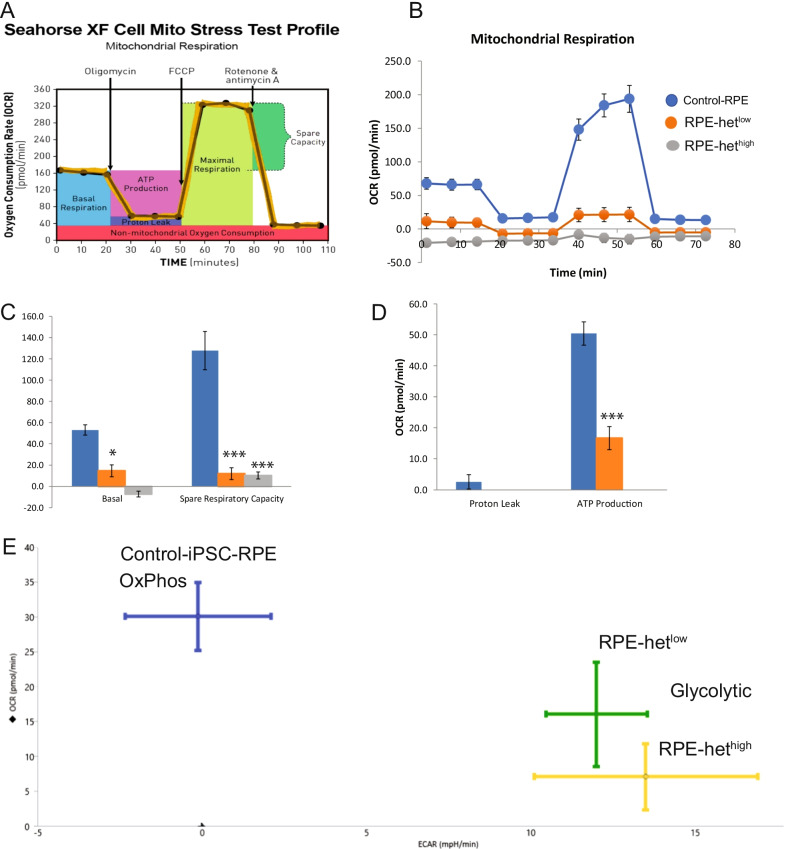
Alterations in mitochondrial bioenergetics in MELAS iPSC-RPE cells. **A** Schematic for the Seahorse XF Cell Mito Stress Test. **B** Mitochondrial oxygen consumption rate (OCR) in control iPSC-RPE, MELAS iPSC-RPE-het^low^, and MELAS iPSC-RPE-het^high^. **C** Changes in basal and spare respiratory capacities. **D** Changes in OCR. **E** Energy map showing changes in OCR and ECAR

Since *p*^0^ cells demonstrate a shift in reliance to alternative bioenergetics (Fig. 2), we investigated the impact of RPE bioenergetics in response to mitochondrial heteroplasmy. Consistent with ARPE-19 data presented in Fig. 2, control-RPE cells showed high OCR (Fig. 8A, B) with concomitant low ECAR (Fig. 8E), showing robust oxidative phosphorylation. However, MELAS-RPE cells had significantly lower basal and spare respiratory capacities with decreased ATP production compared to control-RPE (Fig. 8B–E) . The OCR decreased in MELAS-RPE cells with a corresponding increase in ECAR, indicating a shift from oxidative phosphorylation to aerobic glycolysis. The degree of mitochondrial respiratory depression is consistent with the level of heteroplasmy in MELAS-RPE cells. Thus, MELAS disease modeling reveals defective mitochondrial bioenergetics in RPE cells.

## Discussion

These studies show that iPSC-derived RPE cells can be used as in vitro disease models of mitochondrial dysfunction and potentially applied to improve our understanding of AMD pathology. Such disease models permit characterization of mitochondrial dysfunction and the signaling pathways associated with impaired RPE bioenergetics and provide useful tools for understanding RPE autophagy and mitochondrial recycling. Results from this study provide a roadmap for interrogating RPE mtDNA heteroplasmy by both cellular and molecular methods. The identification of mtDNA mutations present at low level of mutation load in MELAS iPSC-derived RPE cells can be used for fusion of donor platelets to successfully restore mitochondrial function. This approach would alter the balance of heteroplasmy to generate phenotypically “healthy” RPE cells. Alternatively, MELAS iPSC-derived RPE cells with low heteroplasmy can be differentiated into phenotypically “healthy” RPE cells by modulation of mitophagy. These strategies provide a conceptual paradigm for generating healthy RPE cells from patients with mitochondrial diseases through autologous transplantation of iPSC-derived RPE cells that have undergone selection to reduce native pathogenic heteroplasmy.

The RPE transports glucose from the blood to the photoreceptors where glucose is used to produce energy and the by-product lactate is transported to the RPE and used for oxidative phosphorylation and ATP generation [61]. Since mitochondria are the site for oxidative phosphorylation, mitochondria dysfunction in the RPE reduces ATP production. As a result, RPE relies on glycolysis to maintain the cell’s energy requirement, thereby reducing the flow of glucose to the photoreceptors [61]. Our data demonstrate that mitochondria depletion in RPE cells fosters aerobic glycolysis for energy production.

We generated primary cultures of RPE from MELAS-iPSCs with low and high mitochondrial heteroplasmy, as in vitro disease models of degenerative RPE diseases. Previous disease modeling studies using MELAS iPSCs revealed functional and structural defects in RPE cells including impaired phagocytosis of photoreceptor outer segments [26]. Patient-specific iPSC-RPE cells were used to investigate retinal diseases including AMD and to test efficacy of drugs responsible for maintaining mitochondrial function [62]. These studies support the use of patient iPSC-RPE cells to investigate the underlying mechanisms causing the AMD phenotype and to evaluate the efficacy of drugs for improving RPE health. Our iPSC-derived RPE cultures retain many of the phenotypic characteristics of RPE in vivo, including cobblestone morphology, the presence of pigment, TEER (when supplemented), and integral RPE proteins. However, there were striking differences in the bioenergetic profiles of two major pathways, mitochondrial OxPhos and glycolysis. MELAS-derived-RPE cells showed reduced OxPhos and increased glycolysis, thus modeling bioenergetic crisis in the RPE. The MELAS mtDNA mutation has been shown to induce an OxPhos enzyme deficiency [63]. In primary RPE cultures from AMD donors, enhanced glycolysis contributed to ATP production [40]. This cell-specific shift in reliance on alternative bioenergetics has been shown to cause the death of both photoreceptors and RPE, driving AMD pathology [7].

In contrast to the neural retina, the metabolically active RPE utilizes beta-oxidation of fatty acids to meet its energy needs [64]. The RPE cells phagocytose and degrade shed photoreceptor outer segments (POS) leading to the generation of excess RPE lipids. Given that the mitochondrial fatty acid beta-oxidation is critically linked to electron transport, non-metabolized lipids will accumulate in cells lacking functional mitochondria. This may in part account for the increased lipid accumulation in basal deposits and the role of the lipid transport protein APOE as a risk factor for AMD [65]. Our results demonstrate that mitochondria depletion increased expression of APOE, confirming a correlation between impaired mitochondrial function and lipid accumulation in RPE cells, which mirror changes observed in AMD pathogenesis. There is ample evidence of APOE immunoreactivity in basal RPE deposits and drusen; therefore, we postulate that accumulation of dysfunctional mitochondria by the aging RPE contributes to drusen accumulation. Additional studies are necessary to understand the contribution of mitochondrial dysfunction to APOE accumulation and loss of RPE homeostasis.

Our data show that mitochondrial depletion activates STAT3, inhibits AMPK*α* activity, and increases PGC-1*α* expression in *p*^0^ RPE cells. These cellular signaling mechanisms are similar to those observed in MELAS-RPE cells with different levels of heteroplasmy. AMPK*α* activation enhances glycolysis for ATP production and acts as a mediator of an adaptive response to energy deficiency in MELAS cells [66]. Inhibition of AMPK*α* signaling promotes a metabolic shift marked by increased glycolysis and anabolic metabolism [67]. Consistent with this, our data demonstrate that AMPK*α* activity is inhibited in MELAS-RPE cells, which may be associated with disruption of mitochondrial homeostasis. We found that MELAS heteroplasmy increased PGC-1*α* expression and the extent of its upregulation is dependent on the level of heteroplasmy. Overall, our data position AMP-PGC-1*α* signaling as an important regulator of metabolic balance under both low- and high-heteroplasmy conditions.

In *p*^0^ and MELAS-RPE cells, AMPK*α *inhibition and STAT3 activation were correlated with inhibition of autophagic flux. This is consistent with previous studies showing decreased ROS and impaired ROS-dependent AMPK*α* signaling causing reduced autophagosome formation in mtDNA-depleted cells [68, 69]. While perturbations of mitochondrial energy metabolism have been shown to increase pro-survival autophagy, RPE cells lacking mtDNA and those expressing MELAS heteroplasmic mtDNA were autophagy deficient. These observations are consistent with earlier studies showing impairment of mitophagy with marked elevation of ROS, depolarization of mitochondrial membrane potential, and decreased cellular bioenergetics in MELAS iPSCs [33]. The diminished autophagic activity in MELAS-RPE cells was associated with reduced degradation of LC3-II, as a consequence of cathepsin D dysfunction in lysosomes. We observed elevated Prom1 expression in MELAS-RPE cells with high heteroplasmy suggesting that Prom1 compensates autophagy impairment, to confer cytoprotection and enable the diseased RPE to cope with the burden of mutant mitochondria. The current studies do not answer why Prom1 expression varies between differentiated MELAS-RPE cells and undifferentiated iPSCs. Prom1 expression decreases in differentiated MELAS-RPE cells, which is consistent with earlier observations showing downregulation of Prom1 expression after differentiation of iPSC cells [70]. We have shown that cytosolic Prom1 expression in human RPE cells confers protection from oxidative stress by activating autophagy [19]. The change in the role of Prom1 from a cell surface marker to a cytosolic autophagy-associated protein in RPE cells suggests that it supports the survival of the RPE. Our findings suggest that alterations in RPE mitochondrial bioenergetics also influence cellular autophagy. Restoration of autophagic activity may protect RPE cells from degeneration and apoptosis.

High RPE mtDNA heteroplasmy markedly increased PINK1 synthesis, suggesting that deficiency in degradation in these cells caused an accumulation of mitochondria that were not recycled by mitophagy. PINK1 degradation was inhibited in MELAS-RPE cells, and the extent of degradation correlated with the level of heteroplasmy. During mitophagy, p62 is recruited to bind with ubiquitinated outer membrane proteins of depolarized mitochondria [71], thereby promoting damaged mitochondria clearance [72]. Ubiquitination and degradation pathways act as cytoprotective mechanisms that trigger selective clearance of damaged mitochondria. We observed that the p62 level was barely detectable in *p*^0^ cells and bafilomycin treatment had no further effect. These findings imply prior ubiquitination and degradation of endogenous p62 with severe loss of mitochondrial function in *p*^0^ RPE. The difference in p62 levels between *p*^0^- and MELAS-RPE cells suggests that MELAS-RPE cells may be a physiologically more relevant mitochondrial disease model (in which mitophagy is impaired) compared to the *p*^0^ model in literature.

## Conclusions

AMD is a multifactorial disease. It is challenging to develop *in vitro* models recapitulating key features of AMD pathobiology. Studies have demonstrated that iPSC patient-derived RPE models unveil disease phenotypes and provide valid approaches for patient-specific drug screening. Our novel data demonstrate that MELAS iPSC-derived RPE cells are a valuable in vitro model that replicates salient characteristics of RPE functional deficiencies in aging and macular degeneration. Our findings shed new light on the mechanisms associated with mitochondrial dysfunction in RPE degeneration and support the hypothesis that mitochondrial defects drive AMD pathology. Our experimental model demonstrates that loss of RPE mitochondrial function favors a shift from oxidative phosphorylation to glycolysis in the aging RPE. We observed a defect in mitochondrial recycling in MELAS-RPE cells with concomitant reduction in autophagic activity due to AMPK*α* inhibition and STAT3 activation. Further exploration of mitochondrial bioenergetics and autophagy signaling can accelerate our understanding of RPE degeneration during physiological aging and AMD. In summary, our studies provide a conceptual framework for using stem cell-based regenerative medicine in rescuing and rejuvenating diseased RPE in a variety of clinical phenotypes associated with AMD.

## Supplementary Information

**Additional file 1.** **Fig. 1:** Rescue of mitochondrial function by cybrids. Expression of mitochondrial proteins cytochrome C oxidase subunit 4 (COX4), voltage-dependent anion channel 1 (VDAC1), and prohibitin 1 (PHB1) through the process of mitochondrial depletion and cybrid rescue.

## Data Availability

All data generated and/or analyzed during this study are included in this article. The iPSC-derived RPE cells generated during and/or analyzed during the current study are available from the corresponding author on reasonable request.

## Abbreviations

LC3-I/LC3-II: Microtubule-associated light chain 3-I/-II
Prom1: Prominin-1
MELAS: Mitochondrial encephalomyopathy, lactic acidosis, and stroke-like episodes
iPSCs: Induced pluripotent stem cells
mtDNA: Mitochondrial DNA
EtBr: Ethidium bromide
STAT3: Signal transducer and activator of transcription 3
Baf: Bafilomycin A1
CCCP: Carbonyl cyanide m-chlorophenylhydrazone
AMPK*α*: Adenosine monophosphate-activated protein kinase *α*
APOE: Apolipoprotein E
ATP: Adenosine triphosphate
OCR: Oxygen consumption rate
ECAR: Extracellular acidification rate
TEER: Transepithelial electrical resistance
EVOM: Epithelial volt-ohmmeter
PGC-1α: Peroxisome proliferator-activated receptor-γ coactivator (PGC)-1*α*
RPE: Retinal pigment epithelium
AMD: Age-related macular degeneration
PEG: Polyethylene glycol
SMEM: Modified eagle medium suspension, no calcium, no glutamine
TDE: Trypsin-like dissociation enzyme
qPCR: Quantitative polymerase chain reaction
RDM: Retinal differentiation medium
BEST1: Bestrophin 1
CRALBP: Cellular retinaldehyde-binding protein
Oct4: Octamer-binding transcription factor 4 (Oct4)
Sox2: Sex-determining region Y-box transcription factor 2 (Sox2)
NIC: Nicotinamide
IGF1: Insulin-like growth factor 1
FGF: Fibroblast growth factor
VDAC: Voltage-dependent anion channel
OxPhos: Oxidative phosphorylation
PINK1: PTEN-induced kinase 1
PHB1: Prohibitin 1
COX4: Cytochrome C oxidase subunit 4
