# Supplementary material for: Modeling of mitochondrial bioenergetics and autophagy impairment in MELAS-mutant iPSC-derived retinal pigment epithelial cells
Source: Stem Cell Res Ther. 2022 Jun 17;13:260. doi: 10.1186/s13287-022-02937-6 (PMC9205099; doi:10.1186/s13287-022-02937-6)
Supplement: Supplementary file 1 — Additional file 1. Fig. 1: Rescue of mitochondrial function by cybrids. Expression of mitochondrial proteins cytochrome C oxidase subunit 4 (COX4), voltage-dependent anion channel 1 (VDAC1), and prohibitin 1 (PHB1) through the process of mitochondrial depletion and cybrid rescue. [file 13287_2022_2937_MOESM1_ESM.pdf]

Modeling of mitochondrial bioenergetics and autophagy impairment in MELAS-mutant iPSC-derived retinal pigment epithelial cells

Sujoy Bhattacharya, Jinggang Yin, Weihong Huo, Edward Chaum

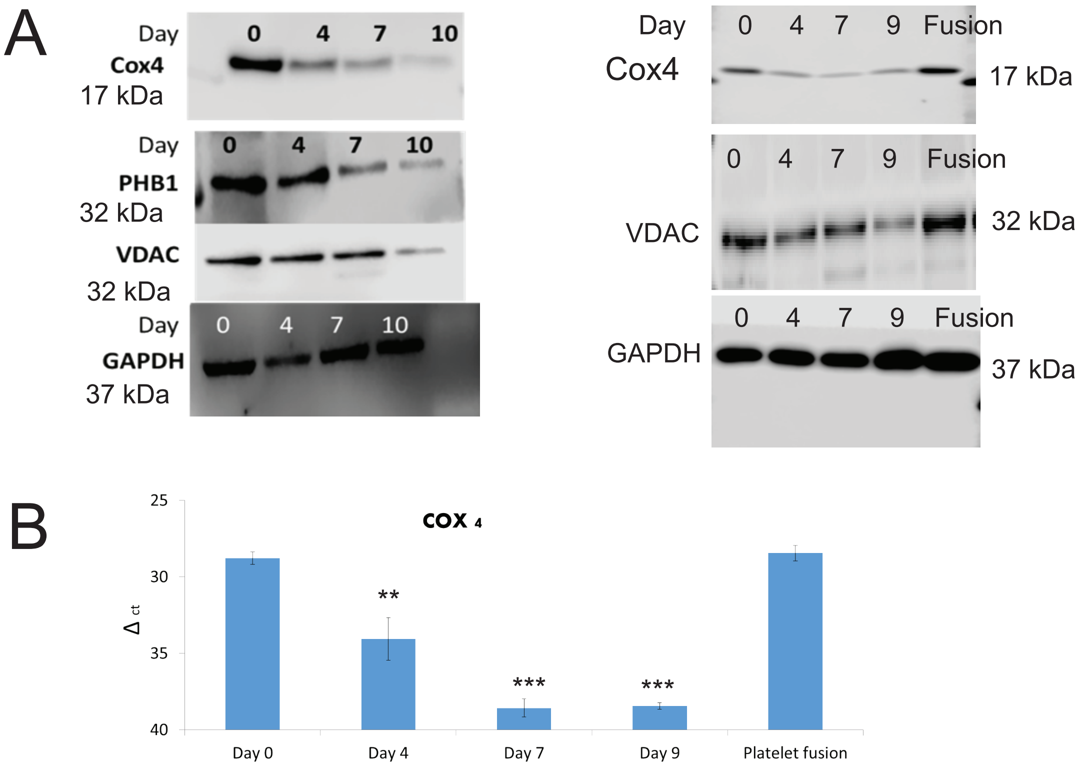

Fig. 1. Rescue of mitochondrial function by cybrids

Expression of mitochondrial proteins cytochome C oxidase subunit 4 (COX4), voltage-dependent anion channel 1 (VDAC1), and prohibitin 1 (PHB1) through the process of mitochondrial depletion and cybrid rescue.
